# Accuracy and Usability of a Novel Algorithm for Detection of Irregular Pulse Using a Smartwatch Among Older Adults: Observational Study

**DOI:** 10.2196/13850

**Published:** 2019-05-15

**Authors:** Eric Y Ding, Dong Han, Cody Whitcomb, Syed Khairul Bashar, Oluwaseun Adaramola, Apurv Soni, Jane Saczynski, Timothy P Fitzgibbons, Majaz Moonis, Steven A Lubitz, Darleen Lessard, Mellanie True Hills, Bruce Barton, Ki Chon, David D McManus

**Affiliations:** 1 Department of Population and Quantitative Health Sciences University of Massachusetts Medical School Worcester, MA United States; 2 Department of Biomedical Engineering University of Connecticut Storrs, CT United States; 3 Division of Cardiology Department of Medicine University of Massachusetts Medical School Worcester, MA United States; 4 Department of Pharmacy and Health Systems Sciences Northeastern University Boston, MA United States; 5 Department of Neurology University of Massachusetts Medical School Worcester, MA United States; 6 Cardiovascular Research Center Massachusetts General Hospital Boston, MA United States; 7 StopAfib.org American Foundation for Women's Health Decatur, TX United States

**Keywords:** mobile health, mHealth, atrial fibrillation, screening, photoplethysmography, electrocardiography, smartwatch

## Abstract

**Background:**

Atrial fibrillation (AF) is often paroxysmal and minimally symptomatic, hindering its diagnosis. Smartwatches may enhance AF care by facilitating long-term, noninvasive monitoring.

**Objective:**

This study aimed to examine the accuracy and usability of arrhythmia discrimination using a smartwatch.

**Methods:**

A total of 40 adults presenting to a cardiology clinic wore a smartwatch and Holter monitor and performed scripted movements to simulate activities of daily living (ADLs). Participants’ clinical and sociodemographic characteristics were abstracted from medical records. Participants completed a questionnaire assessing different domains of the device’s usability. Pulse recordings were analyzed blindly using a real-time realizable algorithm and compared with gold-standard Holter monitoring.

**Results:**

The average age of participants was 71 (SD 8) years; most participants had AF risk factors and 23% (9/39) were in AF. About half of the participants owned smartphones, but none owned smartwatches. Participants wore the smartwatch for 42 (SD 14) min while generating motion noise to simulate ADLs. The algorithm determined 53 of the 314 30-second noise-free pulse segments as consistent with AF. Compared with the gold standard, the algorithm demonstrated excellent sensitivity (98.2%), specificity (98.1%), and accuracy (98.1%) for identifying irregular pulse. Two-thirds of participants considered the smartwatch highly usable. Younger age and prior cardioversion were associated with greater overall comfort and comfort with data privacy with using a smartwatch for rhythm monitoring, respectively.

**Conclusions:**

A real-time realizable algorithm analyzing smartwatch pulse recordings demonstrated high accuracy for identifying pulse irregularities among older participants. Despite advanced age, lack of smartwatch familiarity, and high burden of comorbidities, participants found the smartwatch to be highly acceptable.

## Introduction

### Background

Atrial fibrillation (AF) is the most common heart rhythm problem in the world and the number of patients living with AF is increasing rapidly [1,2]. The arrhythmia confers a 3-fold higher risk for heart failure, a 2-fold risk for dementia, and a 5-fold risk of ischemic stroke among affected individuals, irrespective of the symptom severity or pattern [1,3]. The diagnosis of AF often represents a clinical challenge, especially in its early stages, owing to its paroxysmal and sometimes asymptomatic nature. Despite the fact that short-lived episodes may elude clinical surveillance, brief or infrequent episodes of AF remain associated with higher risk for stroke and death [4,5]. Treatment of AF with oral anticoagulation drastically reduces the risk for stroke by up to 70% [6], but many patients escape detection until after a serious complication. For example, 1 in 5 AF patients present with stroke as their first manifestation of the arrhythmia [7]. New methods for monitoring and screening are needed to facilitate early AF diagnosis, initiate treatment, and reduce suffering and death from the arrhythmia.

Systematic and opportunistic screening of older populations for AF using mobile and digital health is feasible and can identify asymptomatic, community-dwelling individuals with undiagnosed AF [8]. Recent European Society of Cardiology AF guidelines emphasize the importance of opportunistic screening but stop short of recommending systematic screening for all individuals, in part owing to the expensive and sometimes cumbersome nature of the existing rhythm monitoring strategies [9]. There is considerable interest, however, from AF patients and families, AF patient advocacy groups, health care providers, insurers, and health systems to develop lower-cost, more user-friendly solutions for long-term heart rhythm monitoring using mobile health (mHealth) devices to empower patients and reduce suffering from AF [10-12]. The Apple Watch Series 4 recently received Food and Drug Administration (FDA) clearance for its mobile electrocardiogram (ECG) function that has been validated in AF patients (data yet unpublished at the time of writing) [13], marking a monumental milestone in smartwatch AF monitoring and other mobile technology developers are likely to follow suit in the near future. Professional organizations such as the American Heart Association (AHA) have also shown great enthusiasm and support for these ventures, and the current AHA president Dr Ivor Benjamin has claimed that smartwatches that “capture data [...] in real time [are] changing the way we practice medicine” [14]. We and other groups have developed accurate automated, real-time realizable, pulse-based approaches for AF detection using a smartphone, but this approach, as with ECG-based approaches using devices that pair with smartphones (ie, AliveCor Kardia [15]), require that a participant perform an active rhythm check [10,11]. As prior studies have demonstrated that even minutes of AF confer risk for ischemic stroke [16], and as smartwatches can perform passive, frequent pulse assessments over long periods of time [17,18], there is considerable interest in adapting existing pulse-based approaches for use on a smartwatch [17,19,20].

Although appealing, the use of a smartwatch for AF monitoring introduces unique and significant technical challenges, such as motion and noise artifacts generated during activities of daily living (ADLs), as well as usability concerns, as individuals at risk for AF tend to be older, less familiar with mHealth devices, and frequently affected by physical and cognitive impairments that can impede operation of, and comfort with, mHealth technologies [21].

### Objectives

In this investigation, we sought to test the performance of a novel, real-time realizable, automated algorithm for AF discrimination using pulse data obtained from a smartwatch among older individuals with, or at risk for, AF while they executed simulated ADLs. Furthermore, we assessed study participants’ impressions of the smartwatch, generally and across specific usability domains. Finally, we identified characteristics associated with comfort using a smartwatch for rhythm analysis.

## Methods

### Design and Setting

This observational study was designed to evaluate the performance and usability of a smartwatch for heart rhythm analysis among older individuals with, or at high risk for, AF. Participants were enrolled between June 2016 and November 2017 from the ambulatory clinics at the University of Massachusetts Medical Center. All participants provided written informed consent before the study participation. This study was approved by the University of Massachusetts Medical School (UMMS) Institutional Review Board (UMMS IRB number H00009953).

### Study Population

Study staff reviewed the electronic health records (EHR) of all patients presenting for an ambulatory visit. To be considered eligible for enrollment, participants were required to be 21 years of age or older, capable of and willing to provide informed consent, and able to speak and read English. Individuals were excluded from study participation if they were pregnant, incarcerated, had reported an adverse reaction to ECG electrodes or a Holter monitor, or refused to adhere to any aspect of the proposed study protocol, including a brief walk test. The staff telephoned potentially eligible study participants (both those with and without AF) 1 to 2 weeks before their clinic visit to assess interest in study participation. A total of 78 patients were telephoned and 48 patients expressed interest in the study. These patients were then approached for consent after their clinic visit. However, 7 patients declined participation at this time and 1 patient was excluded due to wrist size being too large for the smartwatch, resulting in a final sample of 40 participants.

Trained staff abstracted clinical, electrocardiographic, and laboratory data from the EHR on all participants, including data obtained during the ambulatory visit immediately preceding the study examination. Resting heart rate and rhythm status, as well as vital signs, including respiratory rate, systolic and diastolic blood pressure, and body mass index, were obtained on all participants. The use of cardiovascular medications was also abstracted from the EHR. The wrist circumference and skin tone of the participants were determined by the research staff at the time of their study examination as these factors may potentially influence pulse recordings obtained by the smartwatch.

### Study Procedures to Simulate Activities of Daily Living

**Figure 1:**
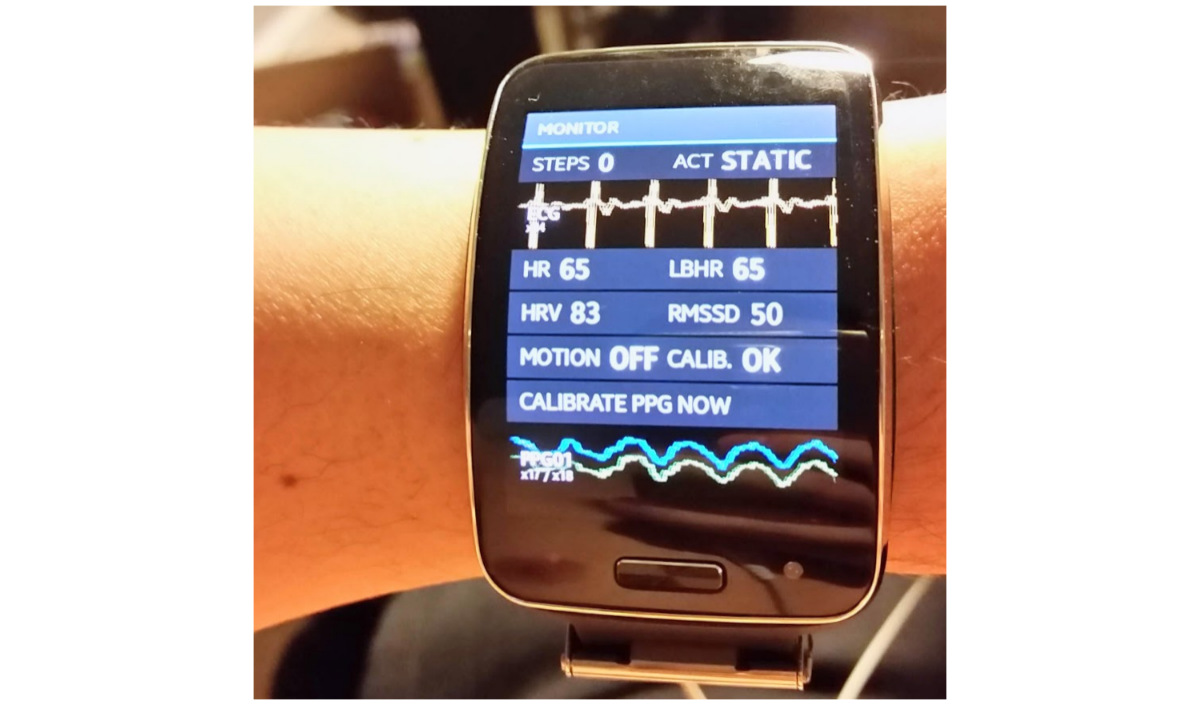
Samsung Simband 2 smartwatch showing simultaneous single-lead ECG (electrocardiogram) and PPG (photoplethysmogram) recordings.

After providing a brief overview of the smartwatch (Samsung Simband 2, Figure 1) and the study protocol, the trained staff helped the participants to don the smartwatch and wear the 7-lead Holter monitor (Rozinn RZ153+ Series, Rozinn Electronics Inc). After ensuring that the watch and Holter monitor were properly fitted and actively recording, participants were asked to sit still for 2 min, followed by a 2-min slow walk (2 miles per hour) down the clinic hallway, followed by a 30-second standing period. The study participants were then instructed to walk quickly (4 miles per hour) down the clinic hallway for 2 min, followed by, in sequence, a 1-min stand, a 1-min period of vertical watch-arm movements, a 1-min period of watch-arm wrist movements, and a 30-second period of standing still [22]. Participants were then asked to sit and stand repeatedly from a chair over a 1-min period. Participants were then asked to climb and descend stairs over a 2-min period, followed by a 1-min period where participants sat and performed deep breathing exercises [23,24]. Slight modifications to the protocol were made for 3 participants to ensure safety: the first participant omitted the repeated sit and stand sequence because of recent knee injury, the second only completed 1 min of climbing stairs because of exertional dyspnea, and the third participant declined the stairs climbing portion of the protocol because of injury from a recent motor vehicle accident. Participants also wore the watch while completing the questionnaire.

### Signal Acquisition, Transfer, and Blinding Procedures

**Figure 2:**
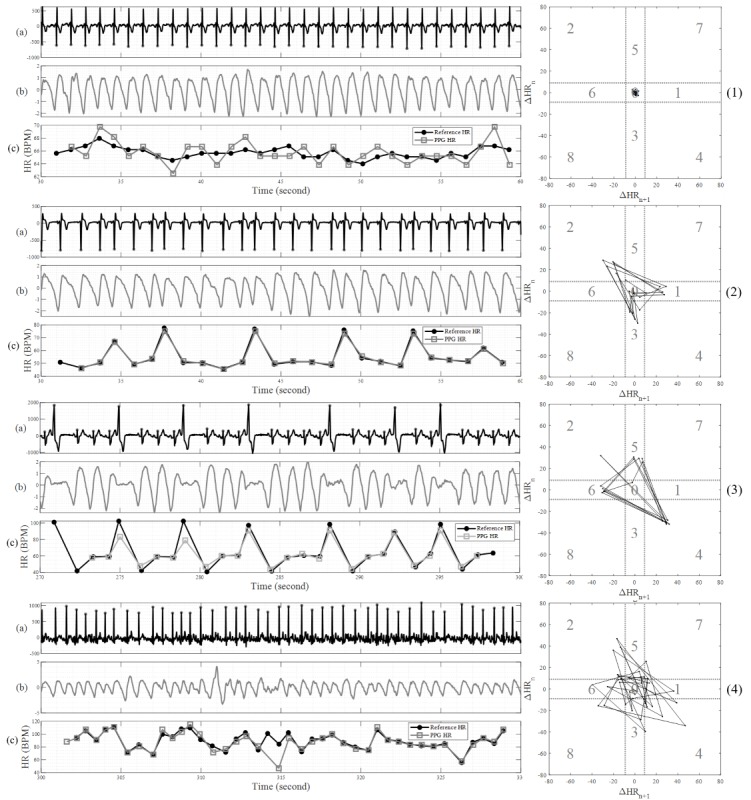
Representative electrocardiographic, pulse, and pulse interval waveforms as recorded by the Samsung Simband 2.0 smartwatch from study participants in various rhythm states. Panels 1 to 4 (top to bottom) represent patients in various rhythm states: (1) shows a patient in normal sinus rhythm, (2) shows a patient with premature atrial contractions, (3) shows a patient with premature ventricular contractions, and (4) shows a patient in atrial fibrillation. For each patient, the single-lead electrocardiogram (a) and pulse plethysmography waveforms (b) are collected by the smartwatch. The pulse interval (c) and Poincare plots (graphed on right) for each patient are also calculated and represented. BPM: beats per min; HR: heart rate; ECG: electrocardiogram; PPG: photoplethysmogram.

The Samsung Simband 2 (Figure 1) is a wrist-worn mHealth device capable of performing continuous real-time monitoring of biophysical data, providing real-time user feedback and wireless, secure, and asynchronous signal transfer [25]. When connected to a secure wireless internet network, the Samsung Simband 2 passively uploads recorded data to ARTIK Cloud (Samsung Electronics), a Web-based cloud-based data storage platform used for research. For this study, Samsung engineers provided us with access to ARTIK Cloud and technical support. We generated a study identification number (ID) for each participant and entered this into the Simband 2 to link photoplethysmogram (PPG) pulse data to secure study data (Figure 2). PPG data identifiable only based on study ID was uploaded to the ARTIK Cloud using a secure connection. Investigators at the University of Connecticut (UConn) group who were blinded to the participants’ rhythm status performed offline rhythm analysis using the real-time realizable algorithm.

### Motion Noise Detection

Once downloaded onto secure UConn study servers, each participant’s pulse data were divided into 30-second segments and then analyzed using a novel motion noise artifact (MNA) detection algorithm. The details of the MNA detection algorithm have been described elsewhere [26]. In brief, the MNA algorithm employs a combination of features derived from a time-frequency analysis and the significance of the accelerometer data’s amplitude to determine MNA severity and a previously derived threshold MNA value is used to determine whether or not a segment is analyzable [26]. After discarding MNA-corrupted PPG segments, we calculated features to classify AF from normal sinus rhythm (NSR).

### Pulse Analysis and Rhythm Discrimination

AF is characterized by disorganized atrial electrical activity that stimulates the ventricles in a random fashion that increases beat-to-beat variability. Our approach to pulse analysis from PPG data has been described in detail and uses 2 validated statistical techniques. The first of these methods, sample entropy (SampEn), measures the complexity of pulse variability. The second, root mean square of successive difference of RR intervals (RMSSD), quantifies peak-to-peak pulse waveform variability; the pulse peak determination methodology has also been previously described [27]. Next, SampEn and RMSSD are combined into a single parameter (Comb) to discriminate between AF and normal rhythms. The combined parameter yields greater area under the receiver operating characteristic curve than either SampEn or RMSSD. We used the combined threshold value of Comb=0.94. Moreover, as a second step, a novel method based on Poincare plot was used to identify ectopic premature atrial or ventricular contractions (PACs, PVCs; paper under review and personal communication by DH, September 2018). Each 30-second segment of PPG data obtained from the smartwatch was blindly assigned, by the UConn team, a designation of *AF* or *not AF* on the basis of this automated analysis. We have previously derived and validated thresholds for detecting PACs/PVCs using a 2-dimensional Poincare plot, which plots the length of each RR interval against the previous RR interval to visualize the beat-to-beat variability in these waveforms among individuals with an irregular pulse [28]. This graph is used to identify bigeminal, trigeminal, and quadrigeminal patterns, which are frequently observed patterns among patients with PACs and PVCs and are common sources of false positive AF detection [28].

### Gold-Standard Electrocardiogram-Based Rhythm Analysis

A 7-lead Holter monitor was used to record an ECG as the gold standard for comparison. ECG data from each participant were labeled with the participant’s study ID and recorded on an Secure Digital card. Staff downloaded the ECG data and transferred it securely to UConn investigators (KC, DH, SKB), where it was stored on firewall protected servers and analyzed separately from the pulse data from watches. Holter data were obtained for 41 participants, but were unavailable for 1 participant, likely because of inadequate contact of leads.

The ECG data were divided into 30-second segments and linked to PPG data using time-stamps and study IDs. Each segment of ECG data was analyzed by a highly accurate and well validated AF detection algorithm using a combination of time-varying coherence functions and Shannon Entropy (ShE) [29] to establish the rhythm status (gold-standard) for each 30-second time period. This algorithm is currently used by ScottCare CardioView Dx, a diagnostic partner program for Holter and event monitors designed by ScottCare Cardiovascular Solutions. Similar to the pulse waveforms obtained from the smartwatch, a subsequent ectopic beat identification algorithm using a Poincare plot threshold was also applied to the ECG data windows deemed to have irregular pulse. For quality control, a board-certified cardiac electrophysiologist (DDM) blinded to the results of the automated ECG analysis reviewed 10% of the 30-second ECG recordings deemed to be consistent with AF and 10% of those deemed to be sinus rhythm. Consistent with prior reports demonstrating excellent performance for AF detection, there was 100% agreement between clinician and automated ECG rhythm determinations [29].

### Study Questionnaire

We characterized system usability, as well as the psychosocial, cognitive, and sociodemographic characteristics of study participants using validated instruments. We also assessed other factors known to influence perceptions of mHealth devices, including education level, yearly income, employment status, and prior experience with smart devices, as measured by smartphone or smartwatch ownership and social media use.

### Usability

Usability in this study was measured globally and across several usability domains. The Brooke System Usability Scale (SUS) is a widely used and validated 10-item questionnaire that assesses multiple dimensions of usability and has been used to assess prior mHealth devices [30]. Responses to each question were scored using a standardized 5-point Likert scale (1=strongly disagree; 5=strongly agree). Responses were weighted equally and converted to achieve a maximum score of 100. For reference, a score of ≥68 is consistent with high usability [30].

A separate investigator-generated assessment was also administered to measure participants’ perceptions of the smartwatch’s ease of use, its overall importance, privacy concerns related to smartwatch use, perceived fit of the device into daily activities, and comfort with use, as measured by stress associated with using the device. Participants responded to all questions using the same 5-point Likert scale used in Brooke SUS.

### Cognitive Impairment and Mood

We employed 3 validated questionnaires to assess cognitive impairment and mood. The Montreal Cognitive Assessment is a validated tool for screening of mild cognitive impairment and is widely used in clinical settings [31]. It includes 30 items and assesses multiple cognitive domains. The scoring ranges from 0 to 30 and a score <26 is indicative of mild cognitive impairment. The Patient Health Questionnaire–9 is a validated 9-item instrument for screening, diagnosing, and monitoring depression in the clinic [32]. A score of 5 to 9 is indicative of mild, 10 to 14 of moderate, and greater than 15 of severe depression. The Generalized Anxiety Disorder scale is a multipurpose 7-item instrument to describe the severity of the patient’s anxiety symptoms in the past 2 weeks [33]. It is a well-validated tool used to screen for a variety of anxiety related disorders, such as generalized anxiety disorder, panic disorder, and posttraumatic stress disorder. The scores range from 0 to 21, and a score of 5 to 9 is considered mild anxiety, 10 to 14 as moderate anxiety or related condition, and greater than 15 as severe anxiety or related condition. This section of the questionnaire was completed by 22 out of the 40 study participants.

### Data Analysis

We first calculated the proportion of noise-free data segments out of the total number of data segments. We then calculated the appropriate test characteristics, including sensitivity, specificity, and accuracy, to examine the performance of our automated pulse analysis algorithm from 314 noise-free pulse segments for the detection of an irregular pulse consistent with AF compared with the results of a validated algorithm examining contemporaneous 7-lead Holter ECG (criterion standard), using established threshold values of RMSSD, SampEn, and Poincare plot [10,11].

The overall usability of the smartwatch for arrhythmia monitoring was examined using validated Brooke SUS. Unadjusted linear regression was then used to identify patient-level characteristics associated with overall Brooke SUS score, as well as Likert-type scores across several usability domains (system ease of use, system importance to user, system privacy concerns, perceived fit of system into daily activities, and comfort with the system, as measured by stress induced by use). Age, sex, history of coronary artery disease, coronary bypass graft procedure, cardioversion, stroke, education level, smartphone ownership, social media use, cognitive impairment, depression, and anxiety were examined as predictors of these usability outcomes. All analyses were performed in MATLAB 9.1 (MathWorks) and Stata 13 (StataCorp).

## Results

### Participant Characteristics

The characteristics of the 40 participants enrolled in the study are shown in Table 1. The average age of the participants was 71 (SD 8) years, 80% (32/40) were male, and all were Caucasian. About 1 in 3 participants had a history of coronary artery disease, 35% (14/40) had undergone a previous cardioversion, and 23% (9/39) were in AF at the time of their study visit. Cognitive impairment was common (59%, 13/22), but symptoms of mild anxiety (18%, 4/22) and depression (18%, 4/22) were less frequently observed. Most participants were retired (70%, 28/40) and most participants reported an annual income of US $50,000, Can $67,404.25, Aus $71,560 or more. Most participants had achieved a high level of education, with over half having completed college or a graduate degree program. Although more than half of the participants owned a smartphone (55%, 22/40), none owned a smartwatch at the time of the study visit. Participants in AF at the time of their study visit had, on average, higher heart rates, as well as higher rates of prior heart failure, cardioversion, anticoagulant use, and anxiety.

### Pulse Rhythm Analysis

A total of 40 participants wore the smartwatch for an average of 42 (SD 14) min, generating a total of 2538 30-second data segments of pulse waveform recordings, of which 314 were noise-free. All data from 1 participant were corrupted by motion/noise artifact, likely from a poor wristband fit. Furthermore, 63 out of the 314 clean 30-second pulse segments were deemed to be *irregular/consistent with AF* and 251 were determined to be *regular/consistent with normal rhythm*. All windows designated irregular pulse were subsequently subjected to ectopic beat detection, which correctly salvaged 6 windows of benign beat irregularities as such compared with ECG data. Final analysis of the smartwatch-generated pulse segments resulted in 54 windows deemed AF, 6 windows deemed PACs/PVCs, and 248 windows deemed NSR. Compared with the ECG rhythm gold standard, AF was correctly identified from smartwatch pulse data in 54 out of 55 windows, corresponding to a sensitivity of 98.2%. The algorithm correctly identified 254 out of 259 windows as showing either normal rhythm or PACs/PVCs compared with the reference standard, corresponding to 98.1% specificity. The pulse analysis algorithm demonstrated excellent overall accuracy (98.1%) in detecting the presence of AF. The algorithm had a positive predictive value of 91.5% and a negative predictive value of 99.6%.

### Usability Analysis

**Figure 3:**
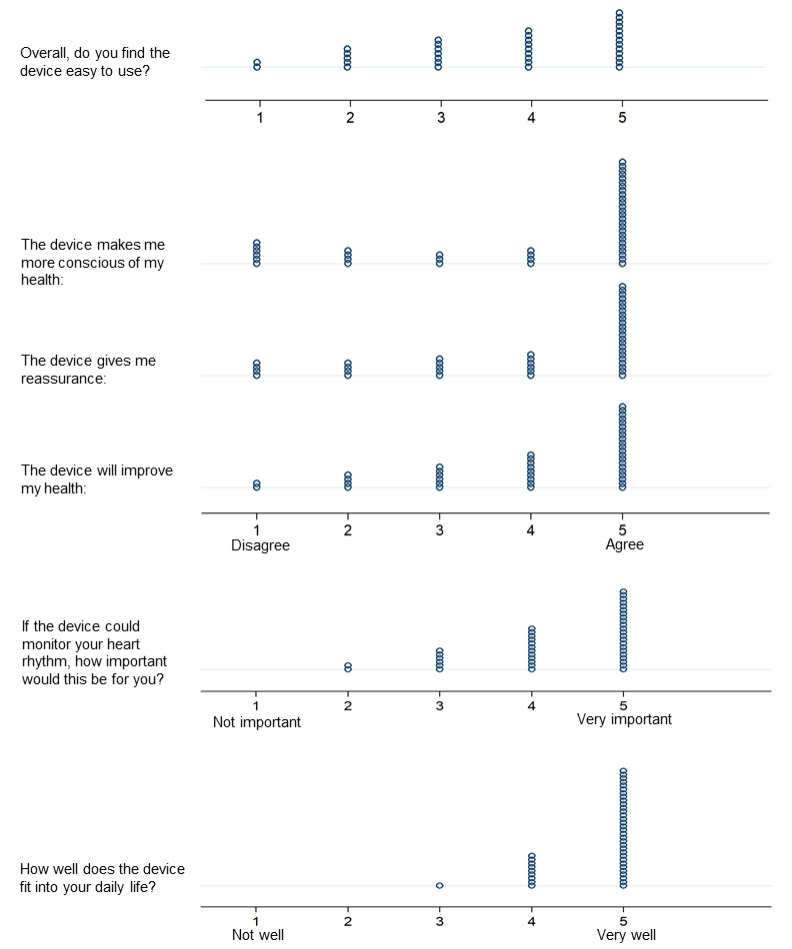
Responses to smartwatch for atrial fibrillation usability questions. Each circle represents an individual participant’s coded response.

All 40 participants were included in the usability analysis. The smartwatch demonstrated high usability for rhythm analysis, as determined using the validated Brooke SUS, with over two-thirds of participants (67.7%) considering the watch to be highly usable. The average Brooke SUS score was 72.9 (SD 17.5). Individual usability domains, including ease of use, importance to user, fit into daily activity, comfort with privacy, and stress associated with use were also assessed using a Likert scale (1 to 5). These usability domains were generated by the investigators for the specific purpose of evaluating this technology and are presented as univariate dot plots to best represent distribution of responses, which may be more meaningful than summary statistics. Overall usability was high across usability domains (Figure 3), with no significant variation across areas. Unadjusted regression analysis (Table 2) showed that older age and prior history of coronary artery bypass surgery were associated with less comfort (as measured by greater stress), when using the smartwatch for rhythm analysis (unadjusted beta coefficients −0.039 and −0.935, respectively). The history of having undergone a cardioversion for AF was associated with greater comfort with sharing rhythm data using the smartwatch (unadjusted beta coefficient 0.72).

**Table 1:** Baseline characteristics of study participants (N=40).

Characteristics			Statistics
**Demographic characteristics**			
	Age (years), mean (SD)		70.6 (8)
	Sex, male, n (%)		32 (80)
	**Race, Caucasian, n (%)**		40 (100)
		Skin tone: tan	27 (68)
		Skin tone: pale	10 (25)
		Skin tone: unspecified	3 (8)
	Wrist circumference, inches, mean (SD), (n=32)		6.9 (0.7)
**Medical characteristics**			
	CHA₂DS₂-VASc score^a^, mean (SD)		2.6 (1.3)
	Body mass index, kg/m^2^, mean (SD)		29.3 (5.1)
	Respiratory rate, bpm^b^, mean (SD)		16.7 (1.2)
	Resting heart rate (per electrocardiogram), bpm, mean (SD)		68.8 (14.3)
	Systolic blood pressure, mm Hg, mean (SD)		126.3 (17.7)
	Diastolic blood pressure, mm Hg, mean (SD)		72.0 (10.4)
	Hypertension, n (%)		25 (63)
	Hyperlipidemia, n (%)		23 (58)
	Current smoking, n (%)		0 (0)
	Diabetes mellitus, Type 2, n (%)		9 (23)
	Coronary artery disease, n (%)		13 (33)
	Prior coronary artery bypass graft, n (%)		7 (18)
	Congestive heart failure, n (%)		2 (5)
	Sleep apnea, n (%)		10 (25)
	Prior cardioversion (%)		14 (35)
	Stroke, n (%)		2 (5)
**Arrhythmia characteristics, n (%)**			
	History of atrial fibrillation		28 (70)
	**Type of atrial fibrillation**		
		Paroxysmal	17 (60)
		Permanent	4 (14)
		Persistent	5 (18)
		Unspecified	2 (7)
	**Rhythm at time of pulse assessment**		
		Sinus rhythm	30 (77)
		Atrial fibrillation	9 (23)
**Treatment characteristics, n (%)**			
	Beta-blocker		27 (68)
	Calcium channel blocker		12 (30)
	Statin		29 (73)
	Antiarrhythmic drug		12 (30)
	Digoxin		1 (3)
	Anticoagulant		21 (53)
**Psychosocial characteristics^c^, n (%)**			
	Cognitive impairment		13 (59)
	Anxiety		4 (18)
	Depression		4 (18)
**Educational status, n (%)**			
	Completed some high school		2 (5)
	Graduated high school		9 (23)
	Graduated high school, some college		5 (13)
	Graduated college		8 (20)
	Graduated college, some graduate school		2 (5)
	Completed a graduate degree		14 (35)
**Employment status, n (%)**			
	Employed full-time		7 (17)
	Employed part-time		4 (10)
	Full time home-maker or caretaker		1 (3)
	Retired		28 (70)
**Income status, n (%)**			
	Less than $10,000		1 (4)
	$10,000 to $29,999		1 (4)
	$30,000 to $49,999		3 (11)
	$50,000 to $69,000		3 (11)
	$70,000 to $89,999		8 (30)
	$90,000 to $149,999		9 (33)
	$150,000 or more		2 (7)
	Unreported		13 (33)
**Technology use, n (%)**			
	Own smartphone		22 (55)
	Own smart watch		0 (0)

^a^CHA₂DS₂-VASc score: clinically used tool for stroke risk assessment.

^b^bpm: beats per minute.

^c^Cognitive impairment is based on Montreal Cognitive Assessment score <26, anxiety is based on Generalized Anxiety Disorder–7 score >4, and depression is based on Patient Health Questionnaire–9 score >4. Data are available for 22 participants for all measures.

**Table 2:** Factors associated with individual usability domains and system usability score.

Variables	Individual usability domains					Overall Brooke system usability score^b^
	Ease of use^b^	Importance to user^b^	Fit into daily activity^b^	Comfort with privacy^b^	Comfort with use^b^	
Age	0.02	−0.01	−0.02	0.00	−0.039^a^	−0.082
Sex, male	−0.38	−0.01	0.14	0.52	−0.063	−8.905
History of coronary artery disease^c^	0.86	0.13	−0.19	−0.23	0.046	−2.680
Prior coronary artery bypass graft^c^	−0.54	−0.04	−0.26	0.21	−0.935^a^	−2.857
Prior cardioversion^c^	−0.22	0.15	0.11	0.72^a^	−0.088	0.238
Prior stroke^c^	1.37	0.27	0.68	0.49	0.842	11.042
Education level^d^	0.04	0.02	0.06	−0.04	0.152	−1.622
Smartphone ownership^c^	−0.07	0.09	0.21	−0.45	0.468	−8.295
Social media use^e^	0.37	−0.02	0.03	−0.06	0.030	−0.814
Cognitive impairment^f^	−0.05	−0.19	−0.17	0.16	−0.188	6.656
Depression^f^	0.37	0.14	0.47	−0.61	−0.158	−6.542
Anxiety^f^	0.21	0.44	0.17	−0.61	−0.158	−8.125

^a^Indicates statistical significance at *P*<.05.

^b^Unadjusted beta coefficients from univariate regression analysis.

^c^Coronary artery disease, coronary artery bypass graft, cardioversion, stroke, smartphone ownership are all coded as yes (1) or no (0).

^d^Education coded from 1 to 6, from completed some high school to some graduate degree.

^e^Social media use coded from 1 to 5, from no use to >6 hours per week.

^f^Cognitive impairment coded as Montreal Cognitive Assessment <26, depression coded as Patient Health Questionnaire–9 >4, anxiety coded as Generalized Anxiety Disorder−7 >4. Cognitive impairment, depression, and anxiety available for 22 participants.

## Discussion

### Principal Findings

We enrolled 40 older patients presenting to an ambulatory clinic in an observational study of mHealth devices for arrhythmia monitoring. Participants wore a smartwatch (the Samsung Simband 2.0, Figure 1) for about 40 min and followed a rigorous protocol to generate motion noise to simulate ADLs. Over 2000 30-second pulse segments were obtained, and after noise elimination, blinded analysis of over 300 30-second pulse segments was conducted using a real-time realizable algorithm. The pulse analysis algorithm demonstrated excellent sensitivity, specificity, and accuracy for the detection of an irregular pulse consistent with AF. Despite their advanced age, lack of familiarity with smartwatches, and high burden of cardiovascular and noncardiovascular comorbidities, study participants found the smartwatch highly acceptable overall and across several important usability domains. We did observe, however, that younger age and prior history of cardioversion were associated with greater comfort with use of the device for heart rhythm monitoring. Our findings demonstrate the accuracy and acceptability of a real-time realizable pulse-analysis algorithm analyzing PPG data from a smartwatch among participants at high risk for incident or recurrent AF.

### Current Atrial Fibrillation Detection Modalities

Early AF detection results in treatment with oral anticoagulants that reduce stroke risk by up to 70% [6], and daily home monitoring for AF among stroke survivors reduces stroke risk by ~18% as compared with conventional in-office ECGs when obtained every 6 to 12 months [34]. In the randomized COMPAS trial, hospitalizations for atrial arrhythmias and stroke were higher in conventionally-monitored patients compared with those prescribed home monitoring, suggesting that home monitoring for AF has clinical impact and is cost effective [34]. Furthermore, in the STROKESTOP study [8], home monitoring using intermittent rhythm recordings identified a significant proportion of previously undiagnosed AF in an older cohort compared with usual care and has been shown in subsequent cost-effectiveness analyses to have a favorable quality-adjusted life years saved [35].

The most commonly prescribed noninvasive ECG monitors (24-hour Holter monitors) demonstrate low yield for AF detection across a wide array of high-risk subgroups, likely as the monitoring coverage of a 24- or 48-hour monitor is simply too brief [36]. For example, the median time to first AF episode was approximately 30 days in the Cryptogenic Stroke and Underlying Atrial Fibrillation study [16,37]. Long-term implantable monitoring significantly improves paroxysmal AF detection rates compared with conventional, shorter-term, noninvasive monitors, but few patients elect to undergo this costly and invasive procedure [16].

Owing to the suspected prevalence and adverse health impact of undiagnosed AF, there is interest in developing new mHealth tools capable of enabling long-term, noninvasive monitoring. Recently, several companies have developed new software and hardware needed to harness the ubiquity and usability of commercial wrist-based wearable devices for AF screening and monitoring [10,12]. AliveCor (AliveCor Inc) recently released the KardiaBand, an FDA-approved, commercially available smartwatch wristband with an embedded ECG electrode designed to pair with the Apple Watch for AF monitoring [38]. The most recent version of this technology aims to use a PPG-based pulse irregularity notification feature to prompt the user to perform an active rhythm check. This is distinct from AliveCor’s Kardia device and other commercial ECG-based devices, such as the MyDiagnostick [39] and Zenicor [40] products, which have no passive monitoring component. A recent study including 100 participants undergoing a cardioversion and wearing the AliveCor KardiaBand showed that, as in our cohort, MNA was common but AF identification was possible and fairly accurate compared with physician review on clean ECG segments [41,42]. Another recent study leveraging the Health eHeart study also demonstrated that an Apple Watch can also be used to collect pulse signals and discriminate AF with modest accuracy compared with self-reported AF using a deep learning neural network approach [19]. This approach has also been shown to be modestly accurate and is computationally intensive. Finally, the SmartWATCHes for Detection of Atrial Fibrillation study, which analyzed pulse data collected from smartwatches using different parameters of heart rate variability than this study (normalized RMSSD and ShE) also found that pulse data can be accurate for AF diagnosis [20]. These promising results, in conjunction with our own, further highlight the importance of addressing human factors such as usability and implementation considerations.

Like the Apple Watch–AliveCor KardiaBand dyad and similar to the recently FDA-cleared Apple Watch 4, the Samsung Simband 2.0 records PPG and single-lead ECG signals using an electrode embedded in the watch (Figure 1). The Samsung Simband platform offered several advantages, leading to its selection for use in our investigation. First, unlike Apple, Samsung provides investigators with unfettered access to a secure data management system designed for research (ARTIK Cloud). Second, Samsung allowed for independent development of open-source apps and data sharing between researchers.

### Pulse-Based Atrial Fibrillation Detection

The pulse analysis approach tested in our study demonstrated high accuracy for the detection of AF using smartwatch pulse recordings despite the fact that participants were asked to perform activities intentionally designed to create MNAs [23,24]. Owing to the intensity of our motion protocols and vulnerability of wrist-based devices to signal corruption, our motion noise detection algorithm deemed 88% of the 30-second pulse segments to be corrupted. Prior investigators have observed similar rates of motion noise corruption when analyzing PPG data for heart rate analysis from smartwatches [43]. The low coverage noted in our study represents a significant potential limitation of PPG-based technologies for heart rhythm monitoring, especially during waking hours and during active periods. This finding highlights the importance of noise detection and the need for algorithms to filter and address MNA [44,45], which may contribute to false positives (owing to noisy segments that are detected as having AF if the algorithms are not sufficiently robust) or false negatives (owing to segments that are eliminated because of noise but include AF if the algorithms are overly restrictive). Despite low rates of pulse coverage, we anticipate higher coverage rates in natural environments, especially during sedentary periods and during sleep [46]. From a diagnostic perspective, although 5 30-second pulse segments were incorrectly identified by the algorithm as being consistent with AF, the clinical significance of such brief episodes is unknown [47]. To date, only episodes lasting 5 min or more have been associated with risk for ischemic stroke [48]. We anticipate that future approaches using pulse data from a smartwatch will be tuned to match performance to clinical use [49], perhaps requiring multiple, sequential 30-second pulse segments to demonstrate pulse irregularity or confirmation with ECG, to reduce false positive AF detection and enhance usefulness.

Our approach to AF detection using a smartwatch involves passive pulse acquisition and real-time analysis using methods that are accurate but not computationally demanding [10,27]. Our approach enhances potential acceptability and successful implementation as it can be feasibly ported into a wide range of smartwatch devices with different hardware specifications, and will not to require any external devices.

### Device Usability and Acceptability

Consistent with this hypothesis, most participants deemed the smartwatch system highly usable overall and expressed comfort with using the system for home heart rhythm monitoring. Our finding that the majority of participants owned a smartphone and were willing to use a smartwatch to monitor themselves debunks the commonly held misconceptions about older Americans at risk for AF and their facility with mobile devices, but is entirely consistent with an emerging literature showing that older Americans are increasing using smart devices and are open to using wearables for disease prevention and treatment [21]. Presently, over 85% of Americans over 65 years currently use mobile phones, a proportion that is increasing [50]. Smartphone use is becoming increasingly common among seniors (10% increase in the last 3 years) [50], and despite well recognized usability barriers, such as physical difficulties, skeptical attitudes, and difficulty learning new technologies, older users can adopt new technologies [51] and often prefer smartphones and smartwatches for running mHealth apps to conventional diagnostic devices, as was observed in our study (Figure 3) [52]. Our results also highlight the importance of proper patient and caregiver education when implementing novel mHealth interventions. Study staff were responsible for set up and deployment of the devices in each participant, which likely facilitated their user experience and contributed to the high perceived usability.

Although we did not find significant associations between any participant characteristics and overall smartwatch usability, we did observe that older age and history of coronary artery bypass graft surgery were associated with lesser comfort using the smartwatch for heart rhythm monitoring, consistent with prior studies [53]. We observed that participants with a history of prior cardioversion for AF were more comfortable using and sharing their heart rhythm data using the smartwatch (Table 2). This finding likely reflects the relative importance of AF monitoring among patients already affected by the disease and perhaps helps to identify an ideal *early adopter* population to test home use of smartwatches for heart rhythm monitoring.

In contrast to prior investigations, our study focused on establishing both the accuracy and usability of a scalable smartwatch-based approach to AF monitoring and screening among a population of older potential users during active periods intended to simulate ADLs. Not only will future studies need to be conducted to examine long-term adherence to smartwatches for AF monitoring among at-risk populations, further work to tune the AF detection algorithm for ideal performance using large, diverse study cohorts will be required. Our findings suggest, however, that older users, when provided support in learning to use smartwatches, can use them well, and that data derived from these devices are of sufficient quality so as to enable high quality rhythm analysis.

### Strengths and Limitations

Our study has several strengths. First, in contrast to most mHealth studies involving younger participants, our study was able to enroll older participants at high risk for incident or recurrent AF. Second, in contrast to other mHealth studies that examine performance of signal processing algorithms from *ideal state* data, we employed a standardized protocol to introduce motion noise to simulate ADLs [23,24]. Third, we conducted blinded analysis of pulse data using validated methods and rigorous quality control [10,11,29]. Finally, despite the rapid development of new technologies for smartwatch-based AF monitoring and enthusiasm in the AF community, we are the first, to our knowledge, to explore usability and acceptability of smartwatches for cardiac monitoring in the elderly.

Several limitations of our study warrant presentation. Our sample was enrolled from an ambulatory clinic at a single tertiary care medical center in central Massachusetts and was relatively homogenous with regard to race and gender. This racial and gender homogeneity significantly limits our ability to generalize these findings to members of other racial groups, community-dwelling individuals not under the care of a physician, or among individuals from other geographic areas. We plan to rectify this and enrich our study population by targeting a more diverse sample in future data collection. Furthermore, the sample was highly educated and reported a relatively high annual income, potentially limiting generalizability to less well-educated or poorer individuals. In addition, our sample was older, affected by a moderate to significant burden of physical and cognitive impairments, and the penetrance of smart device use was lower than the national average [50] (55% owned a smartphone), suggesting that our findings may underestimate the usability of the smartwatch for rhythm analysis in other groups. Our gold standard used was also based on a commercial automated algorithm, and although cardiologist overread performed on 20% of the sample matched the algorithm completely, manual overread on the remaining sample was not performed due to feasibility. In addition, although we intentionally designed study protocols to generate MNA from activities of normal daily life, we did not assess accuracy or usability with home use. The device was tested in a temperature- and light-controlled clinic environment, and disturbance of these factors, in addition to variables unmeasured in our study (such as skin turgor and humidity), may affect performance. Finally, our study population was a convenience sample enriched for participants with AF, and as such, the usability data collected may be affected by selection bias. This inflated AF prevalence also likely resulted in a higher positive predictive value for AF identification than in real-world settings, where the AF prevalence is much lower.

### Conclusions

A novel, real-time realizable software algorithm analyzing pulse data from a smartwatch exhibits excellent performance for the detection of an irregular pulse consistent with AF among older individuals creating motion noise to simulate ADLs. Furthermore, the smartwatch system was deemed highly usable by older participants enrolled in our study, suggesting that long-term monitoring for AF using wrist-based mHealth devices holds promise. Future work is needed to assess provider impressions of the system, to validate findings from our study in much larger and more diverse cohorts, and examine long-term adherence to daily home use, as well as in-field accuracy of AF diagnosis among older individuals at risk for incident or recurrent AF.

## Appendix

Multimedia Appendix 1 Descriptive statistics of participant responses to smartwatch usability assessment.

## Abbreviations

ADLs: activities of daily living
AF: atrial fibrillation
AHA: American Heart Association
ECG: electrocardiogram
EHR: electronic health record
FDA: Food and Drug Administration
ID: identification number
mHealth: mobile health
MNA: motion noise artifact
NIH: National Institute of Health
NSR: normal sinus rhythm
PAC: premature atrial contraction
PPG: photoplethysmogram
PVC: premature ventricular contraction
RMSSD: root mean square of successive difference of RR intervals
SampEn: sample entropy
ShE: Shannon Entropy
SUS: System Usability Scale
UConn: University of Connecticut
UMMS: University of Massachusetts Medical School
