# Supplementary material for: Accuracy and Usability of a Novel Algorithm for Detection of Irregular Pulse Using a Smartwatch Among Older Adults: Observational Study
Source: JMIR Cardio. 2019 May 15;3(1):e13850. doi: 10.2196/13850 (PMC6834225; doi:10.2196/13850)
Supplement: Multimedia Appendix 1 [file cardio_v3i1e13850_app1.docx]

**Supplemental Table 1. Descriptive Statistics of Participant Responses to Smartwatch Usability Assessment**

| **Domain** | **Mean** | **SD** |
| --- | --- | --- |
| Overall, do you consider the heart rhythm detection tool to be easy to use?  *Response: 1 (very difficult to use) – 5 (very easy to use)* | 3.72 | 1.30 |
| Please indicate your agreement with the following statement.  [This device] makes me more conscious of my own health:  *Response: 1 (completely disagree) – 5 (completely agree)* | 3.73 | 1.57 |
| Please indicate your agreement with the following statement.  [This device] gives me reassurance:  *Response: 1 (completely disagree) – 5 (completely agree)* | 3.72 | 1.45 |
| Please indicate your agreement with the following statement.  [This device] will help me be healthier:  *Response: 1 (completely disagree) – 5 (completely agree)* | 3.85 | 1.31 |
| If this smart device could determine your heart rhythm, how important could this monitoring system be for you?  *Response: 1 (not important at all) – 5 (very important)* | 4.36 | 0.81 |
| To what extent do you think the system might fit into your daily life?  *Response: 1 (doesn’t fit at all) – 5 (fits very well)* | 4.74 | 0.50 |
| To what extent do you think the system might cause you to be anxious or stressed?  *Response: 1 (no stress) – 5 (a lot of stress)* | 4.20 | 1.02 |
